# US201 Study: A Phase 2, Randomized Proof-of-Concept Trial of Favipiravir for the Treatment of COVID-19

**DOI:** 10.1093/ofid/ofab563

**Published:** 2021-12-07

**Authors:** Robert W Finberg, Madiha Ashraf, Boris Julg, Folusakin Ayoade, Jai G Marathe, Nicolas C Issa, Jennifer P Wang, Siraya Jaijakul, Lindsey R Baden, Carol Epstein

**Affiliations:** 1 University of Massachusetts Chan Medical School, Worcester, Massachusetts, USA; 2 Houston Methodist Research Institute, Houston, Texas, USA; 3 Ragon Institute of Massachusetts General Hospital, Massachusetts Institute of Technology, and Harvard University, Cambridge, Massachusetts, USA; 4 University of Miami Miller School of Medicine, Miami, Florida, USA; 5 Department of Medicine, Boston University School of Medicine, Boston, Massachusetts, USA; 6 Brigham and Women’s Hospital and Harvard Medical School, Boston, Massachusetts, USA; 7 FUJIFILM Pharmaceuticals USA, Inc., Cambridge, Massachusetts, USA

**Keywords:** favipiravir, COVID-19, hospitalized, human

## Abstract

**Background:**

Favipiravir is used to treat influenza, and studies demonstrate that it has antiviral activity against severe acute respiratory syndrome coronavirus 2 (SARS-CoV-2).

**Methods:**

We performed a randomized, open-label, multicenter, phase 2 proof-of-concept trial of favipiravir in hospitalized adult patients with polymerase chain reaction (PCR)–positive coronavirus disease 2019 (COVID-19). Patients were randomized to standard of care (SOC) or favipiravir treatment (1800mg per os twice a day [b.i.d.] on day 1, followed by 1000mg b.i.d. for 13 days). The primary end point was time to viral clearance on day 29.

**Results:**

Fifty patients were enrolled and stratified by disease severity (critical disease, severe disease, or mild to moderate disease). Nineteen patients were censored from the event of viral clearance based on being SARS-CoV-2 PCR-negative at the study outset, being PCR-positive at day 29, or because of loss to follow-up. Data from the 31 remaining patients who achieved viral clearance show enhanced viral clearance in the favipiravir group compared with the SOC group by day 29, with 72% of the favipiravir group and 52% of the SOC group being evaluable for viral clearance through day 29. The median time to viral clearance was 16.0 days (90% CI, 12.0 to 29.0) in the favipiravir group and 30.0 days (90% CI, 12.0 to 31.0) in the SOC group. A post hoc analysis revealed an effect in the subgroup of patients who were neutralizing antibody–negative at randomization. Treatment-emergent adverse events were equally distributed between the groups.

**Conclusions:**

We demonstrate that favipiravir can be safely administered to hospitalized adults with COVID-19 and believe that further studies are warranted.

**ClinicalTrials.gov registration:**

NCT04358549.

Favipiravir, a nucleoside analog, is active against a broad spectrum of RNA viruses. The drug directly interacts with viral polymerases to inhibit viral RNA transcription without affecting host polymerases. A major advantage of this drug is that it can be orally administered and sustainable serum levels achieved. Over 40 clinical trials for favipiravir have been conducted, and it is licensed in Japan to treat influenza virus that is unresponsive to other agents.

In influenza studies, in vitro treatment with favipiravir leads to inaccurate transcription of the virus, resulting in virus extinction via the accumulation of multiple detrimental mutations. Such “mutational meltdown” means that escape mutants are unlikely to occur and has been demonstrated in both in vitro and in vivo studies with influenza [1, 2]. Importantly, in vivo and in vitro studies with influenza indicate that favipiravir does not select for resistant strains.

Several studies indicate that favipiravir-ribofuranosyl-5’triphosphate (favipiravir-RTP) can be used against SARS-CoV-2. Favipiravir has been shown to inhibit replication of SARS-CoV-2 both in vitro and in a hamster model of infection [3, 4]. The drug binds to the SARS-CoV-2 RNA-dependent RNA polymerase [5–7] and suppresses viral replication by inhibiting the incorporation of natural nucleosides [5]. In vitro studies demonstrate that favipiravir binds to the SARS-CoV-2 polymerase and could mimic adenine and guanine [6], suggesting that its mechanism of action in inhibiting SARS-CoV-2 is analogous to the way it inhibits influenza. In addition, by affecting transcription fidelity, favipiravir appears to act on SARS-CoV-2 by generating mutations that are disadvantageous to the virus, possibly leading to “mutational meltdown,” similar to its activity on influenza [1, 2, 8].

We performed a phase 2 proof-of-concept trial to define the safety and efficacy of favipiravir in hospitalized patients with COVID-19 at 7 US academic medical centers.

## METHODS

### Study Design

We conducted a phase 2, randomized, open-label, multicenter trial of favipiravir to treat adults hospitalized for COVID-19 (NCT04358549). The study was approved by the institutional review boards at all 7 US academic centers, and all participants provided written informed consent. The study was designed before the availability of other therapies for COVID-19 and was conducted from April 2020 through the end of October 2020. The sample size was calculated based on a report by Cai et al. [9], assuming that the median time for viral clearance would be 4 days during favipiravir treatment and 11 days during standard-of-care (SOC) treatment. As this was an exploratory study to determine if favipiravir is active against SARS-CoV-2, these statistical parameters were chosen to enhance the ability to detect a signal of activity. Patients were randomized within 72 hours of hospitalization and stratified by disease severity: critical disease (requiring high-flow O_2_ but not intubation), severe disease (infiltrates on chest x-ray, oxygen saturation level [SpO_2_]<93% on room air, or requiring O_2_ by face mask or cannula), or mild to moderate disease (SpO_2_>94% and respiratory rate <24 without supplemental O_2_). Favipiravir doses were calculated based on an EC_50_ of 9.7 µg/mL [10]. Data from the influenza trial performed in the United States (US316) demonstrated mean trough levels >20 µg/mL with a dosing regimen of 1800mg b.i.d. on day 1 followed by 800mg b.i.d. thereafter (unpublished). Patients received 1800mg of favipiravir per os b.i.d. on day 1, followed by 1000mg b.i.d. (800mg b.i.d. for patients with Child-Pugh A liver disease). For patients unable to swallow pills, the pills were provided as a slurry in a nasogastric tube. The planned duration of therapy with favipiravir was 14 days, which was continued after discharge. The follow-up period was 46 days after the active treatment phase.

### Patient Population

Hospitalized adults (age 18–80 years) with a SARS-CoV-2 PCR-positive nasopharyngeal or oropharyngeal test (within 72 hours of their hospitalization and within 7 days of the first positive PCR for SARS-CoV-2) were eligible for randomization to favipiravir plus SOC or SOC alone. A later addition to the protocol specified that patients needed to have symptom onset within 10 days of presentation. Patients were excluded if they were taking other antivirals (including remdesivir), taking steroids (except for a topical or inhaled preparation or the equivalent of >10mg of prednisone), receiving immune plasma, or taking immunosuppressive or immunomodulatory drugs (including anticancer drugs, interleukins, interleukin antagonists, or interleukin receptor blockers). Beginning in July 2020 with the report of the results of the Randomized Evaluation of COVID-19 Therapy (RECOVERY) trial, dexamethasone at 6mg daily was permitted. Patients with serious chronic diseases, including moderate or severe hepatic disease, and/or unstable renal, cardiac, pulmonary, neurologic, vascular, or endocrinologic diseases requiring medication dose changes within the last 30 days were excluded, as were patients with glomerular filtration rates >20mL/min or requiring hemodialysis/continuous ambulatory peritoneal dialysis, liver impairment greater than Child-Pugh A, uncontrolled psychiatric disease, or a history of alcohol or drug abuse in the previous 6 months. Patients requiring mechanical ventilators at entry were also excluded.

### Study End Points

The primary end point was time to viral clearance, defined as the time (in days) when negative (or below the lower limit of detection) PCR results for SARS-CoV-2 were obtained from scheduled nasopharyngeal and oropharyngeal swabs.

### Secondary End Points

1. Status of clinical recovery as measured by the study-specific 6-point ordinal scale at day 15
2. Time to clinical recovery was assessed up to 29 days and defined as (a) time (hours) from initiation of study treatment (favipiravir+SOC or SOC alone) until normalization of fever, respiratory rate, and oxygen saturation, and alleviation of cough, sustained for at least 72 hours; or discharge; and (b) normalization and alleviation criteria, defined as fever ≤37.2°C (oral), respiratory rate ≤24/min on room air, oxygen saturation SpO2 >94% on room air, and cough mild or absent on a patient-reported scale (severe, moderate, mild, absent)
3. Clinical effect as measured by the NEWS2 system
4. All-cause mortality
5. Frequency of respiratory progression (per SOC at each site), defined as SpO_2_ ≤94% on room air or partial pressure of oxygen/fraction of inspired oxygen <300 mmHg, and requirement for supplemental oxygen or more advanced ventilator support
6. Time to defervescence (those with fever at enrollment)
7. Time to cough reported as mild or absent (those with cough at enrollment rated severe or moderate)
8. Time to dyspnea reported as mild or absent (on a scale of severe, moderate, mild, or absent, in those with dyspnea at enrollment rated as severe or moderate)
9. Frequency of requirement for supplemental oxygen or noninvasive ventilation
10. Time to SARS-CoV-2 RT-PCR-negative in upper respiratory tract specimen
11. Change in SARS-CoV-2 viral load in upper respiratory tract specimen (assessed by area under viral load curve)
12. Frequency of requirement for mechanical ventilation
13. Safety of favipiravir+SOC vs SOC alone
14. C-reactive protein over time
15. Population PK analysis of favipiravir with assessment of maximum plasma concentration (C_max_), minimum plasma concentration (C_min_), and AUC_(0-24h)_ on days 1, 2, 8, and 14

Points 5, 10, and 11 were assessed daily up to 29 days; points 4, 6–9, 12, and 13 were assessed daily up to 29 days and on day 60 (whenever possible).

### Study Assessments

Evaluations of clinical status by both the 6-point ordinal score and the National Early Warning Score 2 (NEWS2) were performed on days 1, 2, 3, 8, 11, 15, 29, and 45. Nasopharyngeal and oropharyngeal swabs for viral RNA, PCR, and TCID_50_ (50% tissue culture infectious dose) were obtained on days 1, 3, 8, 11, 15, and 29 and combined in 1 viral transport media tube for each collection day. Blood for antibodies to SARS-CoV-2 was obtained on days 1, 15, and 29. Blood for pharmacokinetic analysis was collected from patients receiving favipiravir pre- and postdose on days 1, 2, 3, 8, 11, and 14.

All virologic assessments for TCID_50_ and quantitative PCR were conducted by a central laboratory (ViroClinics DDL, Rotterdam Science Tower, Marconistraat 16, 3029 AK, Rotterdam, the Netherlands). The presence of viral neutralizing antibodies was tested by mixing patient serum with SARS-CoV-2 before addition to Vero E6 cells and quantitating the dilution of serum required to achieve 80% inhibition of viral growth (lower limit of detection, dilution of 1:8). The lower limit of quantification (LLOQ) dilution of serum was 1:21.

### Statistical Analysis

**Figure 1.**
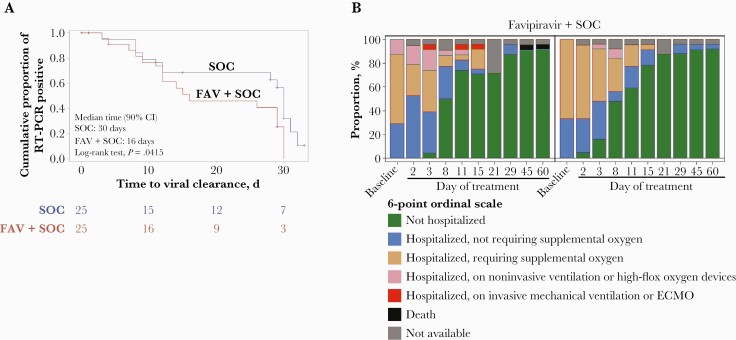
Primary and secondary end points of US201 favipiravir study. A, US201 primary end point (viral clearance). B, Clinical status over time on study-specific 6-point ordinal scale (ITT population). Stacked box plot showing the proportion of subjects in each of the 6 clinical status categories over time for the favipiravir treatment group and the standard-of-care group. Abbreviations: ECMO, extracorporeal membrane oxygenation; FAV, favipiravir; ITT, intention to treat; RT-PCR, reverse transcription polymerase chain reaction; SOC, standard of care.

Statistical analyses were done based on predefined criteria. Using a 2-sided alpha of .10, power of 90%, and a fixed per-subject observation time of 15 days, a sample size of 25 subjects per arm (50 total) was calculated. The primary end point of time to viral clearance (Figure 1) was analyzed with the log-rank test for treatment difference based on a 2-sided alpha level of .10. Intention to treat (ITT) is the primary analytical population and includes all 50 randomized patients. The median time to viral clearance was determined based on the Kaplan-Meier estimator. The time to viral clearance (in days) was defined as the time from randomization to first negative result or lower limit of quantification (LLOQ) result (without any subsequent positive results) as measured by PCR for SARS-CoV-2 via scheduled nasopharyngeal and oropharyngeal swab samples minus the date of first dose+1. Participants were censored if they (1) were PCR-negative in the research lab on day 1, (2) remained PCR-positive on the day 29 visit, or (3) were lost to follow-up without a PCR-negative test. A subset of participants lost to follow-up met criteria of worsened symptoms (defined as an increase of ≥1 point on the 6-point ordinal scale from baseline) on the day of the last PCR measurement or died and were censored at day 29.

**Figure 2.**
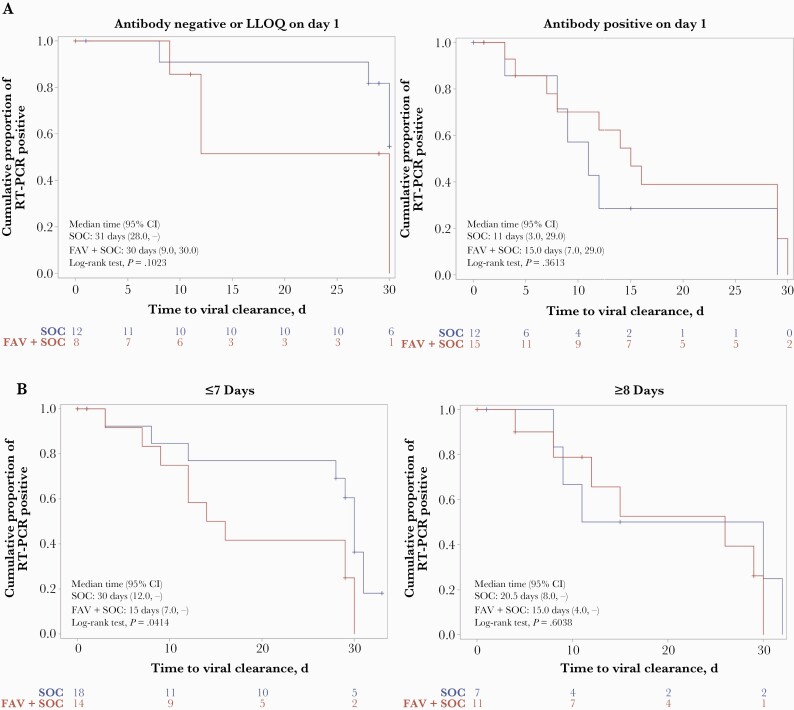
Post hoc subgroup analysis. A, Subgroup analysis by neutralizing antibody at day 1 predose. Time to viral clearance measured in patients with neutralizing antibodies to SARS-CoV-2 that were lower than the LLOQ (left panel) or higher than the LLOQ (right panel). B, Subgroup analysis by days from symptom onset to randomization. Time to viral clearance (by PCR) was measured in patients grouped on the basis of the time between development of COVID-19 symptoms and randomization to the favipiravir plus SOC group or the SOC alone group. Abbreviations: COVID-19, coronavirus disease 2019; LLOQ, lower limit of quantification; PCR, polymerase chain reaction; RT-PCR, reverse transcription polymerase chain reaction; SARS-CoV-2, severe acute respiratory syndrome coronavirus 2; SOC, standard of care.

The total duration of hospitalization was analyzed using an analysis of covariance model. In addition, for this communication, we added 2 post hoc analyses given the emerging literature on baseline SARS-CoV-2 status and viral load clearance kinetics (Figure 2).

Participants with missing data had their clinical status measured by the 6-point ordinal score imputed in the following manner: (1) “not hospitalized” – patients discharged before the visit, (2) “death” – patients who died before the visit, and (3) if there was no evidence of hospital discharge or death, their clinical status was recorded using the multiple imputation method prespecified in the statistical analysis plan.

## RESULTS

### Baseline Patient Characteristics

The median age was slightly higher in the SOC group (58.9 years) than the favipiravir group (55.4 years), and the proportion of patients age ≥65 years was higher in the SOC group (52%) than the favipiravir group (24%). Two patients in the favipiravir group were classified as having critical disease, while no one in the SOC group had critical disease at randomization. Four patients in the favipiravir group required noninvasive ventilation on admission, whereas no one in the SOC group required such support. Other demographic characteristics showed minimal differences between the intent-to-treat groups (Table 1).

**Table 1.** US201 Demographics (ITT Population)

Parameter	Favipiravir+SOC (n=25)	SOC (n=25)	Overall (n=50)
Age, y			
Mean (SD)	55.4 (12.37)	58.9 (13.90)	57.2 (13.14)
Median	58.0	65.0	59.0
Min, max	27, 76	27, 79	27, 79
Sex, No. (%)			
Male	16 (64.0)	14 (56.0)	30 (60.0)
Female	9 (36.0)	11 (44.0)	20 (40.0)
Race, No. (%)			
American Indian or Alaskan Native	0	0	0
Asian	1 (4.0)	0	1 (2.0)
Black or African American	4 (16.0)	6 (24.0)	10 (20.0)
Pacific Islander	0	0	0
Mixed or other	3 (12.0)	7 (28.0)	10 (20.0)
White	17 (68.0)	12 (48.0)	29 (58.0)
Ethnicity, No. (%)			
Hispanic or Latino	13 (52.0)	14 (56.0)	27 (54.0)
Not Hispanic or Latino	12 (48.0)	10 (40.0)	22 (44.0)
Unknown	0	1 (4.0)	1 (2.0)
Smoking status, No. (%)			
Nonsmoker	17 (68.0)	14 (56.0)	31 (62.0)
Ex-smoker	6 (24.0)	11 (44.0)	17 (34.0)
Smoker	2 (8.0)	0	2 (4.0)
Disease severity, No. (%)			
Critical disease	2 (8.0)	0	2 (4.0)
Severe disease	13 (52.0)	15 (60.0)	28 (56.0)
Mild/moderate disease	10 (40.0)	10 (40.0)	20 (40.0)
Any respiratory assistance needed at baseline, No. (%)			
Yes	17 (68.0)	18 (72.0)	35 (70.0)
No	8 (32.0)	7 (28.0)	15 (30.0)
Type of respiratory assistance needed at baseline, No. (%)			
Supplemental oxygen	15 (60.0)	18 (72.0)	33 (66.0)
Noninvasive ventilation	4 (16.0)	0	4 (8.0)
Mechanical ventilation	0	0	0
ECMO	0	0	0
Antibodies to SARS-CoV-2 at baseline, No. (%)			
Yes	15 (60.0)	12 (48.0)	27 (54.0)
No	8 (32.0)	12 (48.0)	20 (40.0)
Unknown	2 (8.0)	1 (4.0)	3 (6.0)
Baseline oxygen saturation level (SpO_2_), No. (%)			
≥96% or n/a	9 (36.0)	9 (36.0)	18 (36.0)
94%–95%	6 (24.0)	8 (32.0)	14 (28.0)
92%–93%	9 (36.0)	6 (24.0)	15 (30.0)
≤91%	1 (4.0)	2 (8.0)	3 (6.0)
Days from symptom onset to randomization			
Mean (SD)	8.4 (6.19)	6.8 (4.69)	7.6 (5.50)
Median	7.0	6.0	6.5
Min, max	1, 28	1, 24	1, 28
Days from latest PCR positivity to randomization			
Mean (SD)	2.0 (0.68)	2.2 (0.75)	2.1 (0.71)
Median	2.0	2.0	2.0
Min, max	1, 4	1, 4	1, 4
Days from start of hospitalization to randomization			
Mean (SD)	2.0 (0.65)	2.0 (0.87)	2.0 (0.76)
Median	2.0	2.0	2.0
Min, max	1, 4	1, 4	1, 4

Two subjects within the favipiravir+standard-of-care treatment group received supplemental oxygen followed by noninvasive ventilation at baseline. They are included in both categories. Values reported as below the lower limit of quantification are characterized as “no.”

Abbreviations: ECMO, extracorporeal membrane oxygenation; ITT, intention to treat; PCR, polymerase chain reaction; SARS-CoV-2, severe acute respiratory syndrome coronavirus 2; SOC, standard of care.

### Efficacy

#### Primary End Point: Time to Viral Clearance

The median time to viral clearance was 16.0 days (90% CI, 12.0 to 29.0) in the favipiravir group and 30.0 days (90% CI, 12.0 to 31.0) in the SOC group (log-rank test, *P*=.0415) (Figure 1). Table 2 illustrates the probability of clearance by time for each group. Nineteen of 50 patients were censored from the study based on predefined criteria described in the “Statistical Analysis” section.

**Table 2.** Time to Viral Clearance by RT-PCR, ITT Population

Parameter	Favipiravir+SOC (n=25)	SOC (n=25)
No. of patients censored,^a^ No. (%)	7 (28.0)	12 (48.0)
No. of patients with viral clearance events, No. (%)	18 (72.0)	13 (52.0)
Time to viral clearance, 75th percentile, d	30.0	31.0
(90% CI)	(26.0 to 30.0)	(30.0 to NE)
Median^b^	16.0	30.0
(90% CI)	(12.0 to 29.0)	(12.0 to 31.0)
25th percentile	12.0	11.0
(90% CI)	(4.0 to 14.0)	(8.0 to 29.0)
Min, max	3, 30	3, 32

Abbreviations: ITT, intention to treat; PCR, polymerase chain reaction; RT-PCR, reverse transcription polymerase chain reaction; SOC, standard of care.

Analysis at day 29. Favipiravir arm censoring: 1 patient had a negative PCR at day 1, 0 patients were PCR-positive at day 29, 4 patients were lost to follow-up with failure to become PCR-negative at last assessment, and 2 patients had worsened symptoms on the day of the last PCR measurement and were censored at day 29. SOC arm censoring: 1 patient had a negative PCR at day 1, 3 patients were PCR-positive at day 29, 6 patients were lost to follow-up with failure to become PCR-negative at last assessment, and 2 patients had worsened symptoms on the day of the last PCR measurement and were censored at day 29.

*P*=.0415, log-rank test.

#### Explanation of Censorship of the Primary End Point

Nineteen patients were censored based on predetermined criteria. Two (1 in the SOC group and 1 in the favipiravir group) were PCR-negative at day 1 in the research laboratory, despite being PCR-positive before randomization at the clinical laboratory, 3 (3 in the SOC group and 0 in the favipiravir group) were still PCR-positive on day 29, and 14 (8 in the SOC group and 6 in the favipiravir group) were lost to follow-up. Of the 14 who were lost to follow-up, 4 (2 in the SOC group and 2 in the favipiravir group) met the criteria of worsened symptoms and were censored on day 29, instead of the day of the patient’s last PCR assessment (Table 2).

#### Secondary End Points

1) **Status of clinical recovery based on the 6-point ordinal scale up to day 60.** The favipiravir group had a lower score than the SOC group on day 8 (odds ratio [OR], 2.388; 90% CI, 0.912 to 6.257) but a higher score than the SOC group at day 15 (OR, 0.684; 90% CI, 0.208 to 2.246) (Figure 1; Supplementary Table 1). Three of the 4 patients in the favipiravir group receiving noninvasive oxygen at randomization improved without the subsequent need for O_2_. The median time to clinical recovery was 7.5 days (90% CI, 4.0 to 9.0) in the favipiravir group and 6.5 days (90% CI, 4.0 to 9.0) in the SOC group, with no significant differences found.
2) **Time to aggregate NEWS2 score of ≤2 or discharge.** The median time to aggregate NEWS2 score of ≤2 was 4.0 days (90% CI, 3.0 to 8.0) in the favipiravir group and 7.0 days (90% CI, 4.0 to 10.0) in the SOC group, with no significant difference noted between the groups (Supplementary Table 2).
3) **Total duration of hospitalization.** The number of days of hospitalization was greater in the favipiravir group than the SOC group (least squares mean difference, 3.2; 90% CI, –2.1 to 8.5; but no significant difference was found) (Supplementary Table 3). One patient in the favipiravir group had diabetes, asthma, hyperlipidemia, and hypertension; her hospital course was complicated by intubation and mechanical ventilation, as well as fungemia. Her prolonged period of hospitalization (>60 days) was attributed to significant comorbidities. After excluding this patient from the analysis, the difference in the mean length of stay of the hospitalization period was shortened to 0.6 days.

### Safety

Fifteen patients in the favipiravir group and 19 patients in the SOC group had adverse events (Table 3). The most common adverse events were increased gamma-glutamyltransferase (12.5%; 3/25) and acute kidney injury (12.5%; 3/25) in the favipiravir group and an increase in inflammatory markers (16%; 4/25) and respiratory failure (16%; 4/25) in the SOC group. Supplementary Table 4 shows adverse events by MedDRA System Organ Class. Three serious adverse events were seen in the favipiravir group (none related to the drug), and 4 serious adverse events were seen in the SOC group (Supplementary Table 5). No deaths occurred in the SOC group. The 1 death in the favipiravir group occurred after discharge and was related to a cerebral bleed thought to be unrelated to either COVID-19 or favipiravir.

**Table 3.** US201 Treatment-Emergent Adverse Event Summary (Safety Population)

Parameter	Favipiravir+SOC (n=24), No. (%)	SOC (n=25), No. (%)	Overall (n=49), No. (%)
≥1 TEAE	15 (62.5)	19 (76.0)	34 (69.4)
≥1 TEAE related to study drug	5 (20.8)	0	5 (10.2)
≥1 grade ≥3 TEAE	2 (8.3)	4 (16.0)	6 (12.2)
≥1 grade ≥3 TEAE related to study drug	0	0	0
≥1 SAE	2 (8.3)	3 (12.0)	5 (10.2)
≥1 SAE related to study drug	0	0	0
≥1 TEAE leading to study discontinuation	0	2 (8.0)	2 (4.1)
≥1 TEAE leading to death	1 (4.2)	0	1 (2.0)

Abbreviations: SAE, serious adverse event; SOC, standard of care; TEAE, treatment-emergent adverse event.

### Pharmacokinetic Data

Mean plasma favipiravir levels of 34.0, 33.9, 22.1, and 42.4 µg/mL were achieved postdosing at days 1, 3, 8, and 11 in 19 patients tested (only a small number of samples were available at day 8). Predose mean levels ranged from 3.6 to 21.3 µg/mL (Supplementary Table 6). Levels of T-705 M1 (a metabolite of favipiravir) were highest on day 1 (mean, 15.1 µg/mL), and means ranged from 3.4 to 6.6 µg/mL (Supplementary Table 6). Area under the curve (AUC)/24 hours ranged from a median of 414 µg/h/mL on day 1 to 786 on day 8 and 772 on day 14 (Supplementary Table 7).

### Post Hoc Analyses

#### Time to Viral Clearance Based on Neutralizing Antibody Titers

A post hoc analysis of time to viral clearance (measured by PCR) revealed that the effect of favipiravir was seen in patients with low levels of neutralizing antibody at baseline and not in patients with higher levels of neutralizing antibody. Neutralizing antibody levels were measured on day 1, before the first dose of favipiravir (or SOC). Subjects were divided into those with neutralizing antibodies at the LLOQ (Figure 2A, left) and those with higher levels (Figure 2A, right). Favipiravir treatment resulted in more rapid clearance of virus (measured by PCR) in the antibody-negative patients. There was no benefit of favipiravir in the antibody-positive group.

#### Time to Viral Clearance Based on Onset of Symptoms

To determine where favipiravir might be most useful clinically, a post hoc analysis of time to viral clearance (by PCR) was conducted based on the number of days between the development of symptoms and the first day of treatment (Figure 2B). The data indicate that there was no positive effect of favipiravir in patients randomized after 8 days of symptoms, while patients randomized at ≤7 days had enhanced viral clearance when favipiravir was added to SOC.

## DISCUSSION

We report the first randomized phase 2 trial of favipiravir for COVID-19 hospitalized patients performed in the United States. In our study, favipiravir enhanced clearance of SARS-CoV-2 compared with SOC as measured by PCR. We show that favipiravir shortens the duration of viral shedding, the primary end point of the study, suggesting that the drug could be best used either prophylactically or early during infection. Early use of this oral medication for mild disease would seem to be the optimal indication. Although the conclusions from this “proof-of-concept” study are limited by the study’s small size, the heterogeneity of patients with COVID-19, and the delay in treatment after onset of symptoms, our results provide data facilitating the design of future clinical trials to test favipiravir for the treatment of SARS-CoV-2.

A recent meta-analysis of 145 favipiravir studies on COVID-19 showed that favipiravir leads to a higher rate of viral clearance, defervescence, chest computed tomography (CT) improvement, and hospital discharge [11]. A more recent study of hospitalized patients was carried out in Japan [12]. In this study of patients with COVID-19 pneumonia, but with baseline SpO_2_ values ≥94%, patients receiving favipiravir had more rapid clinical improvement and significantly shorter time to viral clearance compared with the placebo group. The researchers noted a larger effect of treatment in patients whose symptom onset was within 5 days of treatment. Consistent with the data reported here, post hoc analysis of the JP324 data indicated that the group that was IgA and IgG antibody negative at predose had a better response to treatment with favipiravir than those who already had antibodies to the virus. A larger, multicenter, randomized, double-blind study of the use of favipiravir in high-risk hospitalized patients with early-onset COVID-19 is currently underway in Japan.

COVID-19 is generally thought to be a biphasic disease, with the initial events related to the virus attacking the host cells, while the later effects are generally thought to result from dysfunctional host responses. Favipiravir is effective when given before exposure to SARS-CoV-2 in hamsters. A recent study by Drouich et al. noted that in hamsters infected with SARS-CoV-2, favipiravir’s efficacy in reducing viral loads was associated with large numbers of mutations in the virus with consequent drops in viral loads [3]. They noted that the plasma levels seen in hamsters were achievable in humans based on prior trials. Here we show that plasma levels above the EC_50_ seen in in vitro studies can be obtained without toxicity and are sustained over the 14-day treatment. The original proposed end point, Tissue Culture Infectious Dose 50 (TCID_50_), was not sensitive enough for assessment, so the outcome was measured by PCR (rated positive or negative by the laboratory).

Our study looked at a heterogeneous group of hospitalized patients, and the vagaries of randomization skewed the favipiravir group toward a younger, but sicker, group compared with the SOC group. Nevertheless, the time to viral clearance (the primary end point) was improved in the favipiravir arm, despite the fact that patients in the favipiravir arm were randomized at a mean of 8.4 days after symptom onset (the mean in the SOC group was 6.8 days). Several previous studies have suggested that giving the drug earlier in disease is likely to result in better outcomes, and this could have affected the clinical outcomes in this study. Similar observations have been made for other antivirals for influenza as well as for both antiviral agents (eg, remdesivir) and monoclonal antibodies used in the treatment of SARS-CoV-2 [13, 14].

Our study demonstrates that favipiravir can be given safely to patients with COVID-19 and that clearance of virus is enhanced. In this study (US201), post hoc analysis demonstrated that favipiravir was efficacious only in patients who were antibody negative at day 1. A Japanese study (JP324) conducted with a less severely ill population than ours noted an effect of favipiravir in both antibody-positive and antibody-negative subjects, but the effect was much greater in antibody-negative subjects. Thus, both studies suggest that the most effective strategy is to administer favipiravir before the patient develops natural antibodies and within a week of developing symptoms. As favipiravir can be given orally and has a track record of safety (including this study), our results support using favipiravir prophylactically in people at high risk of developing severe disease (either before or immediately after exposure). We hypothesize that favipiravir could be given to nursing home residents in the event of an outbreak or families or close contacts of people diagnosed with COVID-19. Trials in outpatients are currently underway to test these hypotheses in the United States. Future studies examining prophylactic use in humans at high risk of severe COVID-19 following exposure are indicated.

## Supplementary Material

ofab563_suppl_Supplementary_Materials
