# Supplementary material for: US201 Study: A Phase 2, Randomized Proof-of-Concept Trial of Favipiravir for the Treatment of COVID-19
Source: Open Forum Infect Dis. 2021 Dec 7;8(12):ofab563. doi: 10.1093/ofid/ofab563 (PMC8651156; doi:10.1093/ofid/ofab563)
Supplement: ofab563_suppl_Supplementary_Materials [file ofab563_suppl_supplementary_materials.docx]

**SUPPLEMENTARY MATERIALS FOR:**

**US201 Study: A phase 2, randomized proof-of-concept trial of favipiravir for the treatment of COVID-19**

**Robert W. Finberg^1^, Madiha Ashraf^2^, Boris Julg^3^, Folusakin Ayoade^4^, Jai G. Marathe^5^, Nicolas C. Issa^6^, Jennifer P. Wang^1^, Siraya Jaijakul^2^, Lindsey R. Baden^6^, Carol Epstein^7^**

**Affiliations:**

^1^University of Massachusetts Chan Medical School, Worcester, MA

^2^Houston Methodist Research Institute, Houston, TX

^3^Ragon Institute of Massachusetts General Hospital, Massachusetts Institute of Technology, and Harvard University, Cambridge, MA

^4^University of Miami, Miami-Dade County, FL

^5^Department of Medicine, Boston University School of Medicine, Boston, MA

^6^Brigham and Women’s Hospital and Harvard Medical School, Boston, MA

^7^FUJIFILM Pharmaceuticals U.S.A., Inc., Cambridge, MA

**Running title**: Favipiravir for COVID-19 treatment

**Article type:** Major Article

**Supplemental Table 1**

| **Analysis Visit** | **Favipiravir+SOC (N=25)** | **SOC (N=25)** |
| --- | --- | --- |
| ***Day 1*** |  |  |
| Number of Subjects, n | 24 | 24 |
| Not hospitalized, n (%) | 0 | 0 |
| Hospitalized, not requiring supplemental oxygen, n (%) | 7 (29.2) | 8 (33.3) |
| Hospitalized, requiring supplemental oxygen, n (%) | 14 (58.3) | 16 (66.7) |
| Hospitalized, on non-invasive ventilation or high-flow oxygen devices, n (%) | 3 (12.5) | 0 |
| Hospitalized, on invasive mechanical ventilation or ECMO, n (%) | 0 | 0 |
| Death, n (%) | 0 | 0 |
| Not available, n (%) | 0 | 0 |
| ***Day 2*** |  |  |
| Number of Subjects, n | 20 | 21 |
| Not hospitalized, n (%) | 0 | 1 (4.8) |
| Hospitalized, not requiring supplemental oxygen, n (%) | 10 (50.0) | 6 (28.6) |
| Hospitalized, requiring supplemental oxygen, n (%) | 5 (25.0) | 13 (61.9) |
| Hospitalized, on non-invasive ventilation or high-flow oxygen devices, n (%) | 3 (15.0) | 0 |
| Hospitalized, on invasive mechanical ventilation or ECMO, n (%) | 0 | 0 |
| Death, n (%) | 0 | 0 |
| Not available, n (%) | 2 (10.0) | 1 (4.8) |
| Cumulative Odds Model [1], Odds Ratio = 1.292; Clinical Status at Day 2, 90% CI (0.479, 3.486) | | |
| ***Day 3*** |  |  |
| Number of Subjects, n | 24 | 25 |
| Not hospitalized, n (%) | 1 (4.2) | 4 (16.0) |
| Hospitalized, not requiring supplemental oxygen, n (%) | 8 (33.3) | 8 (32.0) |
| Hospitalized, requiring supplemental oxygen, n (%) | 8 (33.3) | 11 (44.0) |
| Hospitalized, on non-invasive ventilation or high-flow oxygen devices, n (%) | 4 (16.7) | 1 (4.0) |
| Hospitalized, on invasive mechanical ventilation or ECMO, n (%) | 1 (4.2) | 0 |
| Death, n (%) | 0 | 0 |
| Not available, n (%) | 2 (8.3) | 1 (4.0) |
| Cumulative Odds Model [1], Odds Ratio (0.565); Clinical Status at Day 3, 90% CI (0.229, 1.397) | | |
| ***Day 8*** |  |  |
| Number of Subjects, n | 23 | 25 |
| Not hospitalized, n (%) | 12 (52.2) | 12 (48.0) |
| Hospitalized, not requiring supplemental oxygen, n (%) | 6 (26.1) | 2 (8.0) |
| Hospitalized, requiring supplemental oxygen, n (%) | 2 (8.7) | 7 (28.0) |
| Hospitalized, on non-invasive ventilation or high-flow oxygen devices, n (%) | 1 (4.3) | 2 (8.0) |
| Hospitalized, on invasive mechanical ventilation or ECMO, n (%) | 0 | 0 |
| Death, n (%) | 0 | 0 |
| Not available, n (%) | 2 (8.7) | 2 (8.0) |
| Cumulative Odds Model [1], Odds Ratio 2.388; Clinical Status at Day 8, 90% CI (0.912, 6.257) | | |
| ***Day 11*** |  |  |
| Number of Subjects, n | 24 | 22 |
| Not hospitalized, n (%) | 18 (75.0) | 13 (59.1) |
| Hospitalized, not requiring supplemental oxygen, n (%) | 2 (8.3) | 4 (18.2) |
| Hospitalized, requiring supplemental oxygen, n (%) | 1 (4.2) | 4 (18.2) |
| Hospitalized, on non-invasive ventilation or high-flow oxygen devices, n (%) | 1 (4.2) | 0 |
| Hospitalized, on invasive mechanical ventilation or ECMO, n (%) | 1 (4.2) | 0 |
| Death, n (%) | 0 | 0 |
| Not available, n (%) | 1 (4.2) | 1 (4.5) |
| Cumulative Odds Model [1], Odds Ratio 2.736; Clinical Status at Day 11, 90% CI (0.908, 8.241) | | |
| ***Day 15*** |  |  |
| Number of Subjects, n | 25 | 23 |
| Not hospitalized, n (%) | 18 (72.0) | 18 (78.3) |
| Hospitalized, not requiring supplemental oxygen, n (%) | 1 (4.0) | 3 (13.0) |
| Hospitalized, requiring supplemental oxygen, n (%) | 4 (16.0) | 1 (4.3) |
| Hospitalized, on non-invasive ventilation or high-flow oxygen devices, n (%) | 0 | 0 |
| Hospitalized, on invasive mechanical ventilation or ECMO, n (%) | 1 (4.0) | 0 |
| Death, n (%) | 0 | 0 |
| Not available, n (%) | 1 (4.0) | 1 (4.3) |
| Cumulative Odds Model [1], Odds Ratio 0.684; Clinical Status at Day 15, 90% CI (0.208, 2.246) | | |
| ***Day 29*** |  |  |
| Number of Subjects, n | 25 | 25 |
| Not hospitalized, n (%) | 22 (88.0) | 22 (88.0) |
| Hospitalized, not requiring supplemental oxygen, n (%) | 2 (8.0) | 2 (8.0) |
| Hospitalized, requiring supplemental oxygen, n (%) | 0 | 0 |
| Hospitalized, on non-invasive ventilation or high-flow oxygen devices, n (%) | 0 | 0 |
| Hospitalized, on invasive mechanical ventilation or ECMO, n (%) | 0 | 0 |
| Death, n (%) | 0 | 0 |
| Not available, n (%) | 1 (4.0) | 1 (4.0) |
| Cumulative Odds Model [1] Odds Ratio 1.516; Clinical Status at Day 29, 90% CI (0.250, 9.182) | | |
| ***Day 45*** |  |  |
| Number of Subjects, n | 23 | 23 |
| Not hospitalized, n (%) | 21 (91.3) | 21 (91.3) |
| Hospitalized, not requiring supplemental oxygen, n (%) | 0 | 1 (4.3) |
| Hospitalized, requiring supplemental oxygen, n (%) | 0 | 0 |
| Hospitalized, on non-invasive ventilation or high-flow oxygen devices, n (%) | 0 | 0 |
| Hospitalized, on invasive mechanical ventilation or ECMO, n (%) | 0 | 0 |
| Death, n (%) | 1 (4.3) | 0 |
| Not available, n (%) | 1 (4.3) | 1 (4.3) |
| ***Day 60*** |  |  |
| Number of Subjects, n | 25 | 25 |
| Not hospitalized, n (%) | 23 (92.0) | 23 (92.0) |
| Hospitalized, not requiring supplemental oxygen, n (%) | 0 | 1 (4.0) |
| Hospitalized, requiring supplemental oxygen, n (%) | 0 | 0 |
| Hospitalized, on non-invasive ventilation or high-flow oxygen devices, n (%) | 0 | 0 |
| Hospitalized, on invasive mechanical ventilation or ECMO, n (%) | 0 | 0 |
| Death, n (%) | 1 (4.0) | 0 |
| Not available, n (%) | 1 (4.0) | 1 (4.0) |

[1] A proportional odds model is fitted based on the cumulative logit function, with clinical status as dependent variable and treatment group, disease severity, analysis visit and analysis visit/treatment group interaction as independent variables. For a given visit day, individuals in the Favipiravir+SOC treatment group are x.xxxx (see odds ratio by day) more likely to not be hospitalized instead of being hospitalized or dead than those in the SOC treatment group.

**Supplemental Table 2. Time to Aggregate NEWS2 Score**

| **Parameter** | **Favipiravir+SOC (N=25)** | **SOC (N=25)** |
| --- | --- | --- |
| Number of Patients with Aggregate NEWS2 Score ≤2 Events [1], n (%) | 19 (76.0) | 19 (76.0) |
| Time to Aggregate NEWS2 Score ≤2 (days) [2] | 15.0 | 11.0 |
| *75^th^ Percentile* |  |  |
| (90% CI) | (7.0, 26.0) | (9.0, 18.0) |
| Median | 4.0 | 7.0 |
| (90% CI) | (3.0, 8.0) | (4.0, 10.0) |
| *25th Percentile* | 3.0 | 4.0 |
| (90% CI) | (2.0, 3.0) | (2.0, 6.0) |
| Min, Max | 2, 26 | 1, 18 |
| Log-Rank Test, p-value=0.9879 | | |
| Product-Limit Probability [3], % | | |
| 3 days | 57.1 | 76.2 |
| 8 days | 28.6 | 42.9 |
| 11 days | 28.6 | 23.8 |
| 15 days | 23.8 | 12.7 |
| 29 days | 9.5 | 6.3 |
| Number of Patients  Censored, n (%) | 6 (24.0) | 6 (24.0) |

[1] Time to NEW2 score of two or less is defined as the number of days from enrollment until the score of ≤ 2 (without score of 3 or more after that) or discharge.

[2] Quartile estimates from the product-limit (Kaplan-Meier) method, with associated 90% log-log confidence intervals.

[3] Based on the Distribution Function estimates from product-limit method.

**Supplemental Table 3. Total Duration of Hospitalization**

|  | **Favipiravir+SOC (N=25)** | **SOC (N=25)** |
| --- | --- | --- |
| Duration of Hospitalization (days) [1], n | 25 | 25 |
| Mean (SD) | 11.5 (12.60) | 9.6 (7.60) |
| Median | 7.0 | 7.0 |
| Min, Max | 2,60 | 1, 28 |
| **ANCOVA model [2]** |  |  |
| Duration of hospitalization (days) LS Means Difference | 3.2 |  |
| 90% CI (-2.1, 8.5) |  |  |

[1] If the date of discharge is unknown, the duration of hospitalization was considered as 1 day.

[2] An ANCOVA model is fitted, stratified by disease severity, with total duration of hospitalization as dependent variable and treatment group and age as independent variables. The covariate included will be baseline SpO_2_.

**Supplemental Table 4. Adverse Events by MedDRA System Organ Class**

| **System Organ Class Preferred Term, n (%)** | **Favipiravir+SOC (N=24)** | **SOC**  **(N=25)** | **Overall**  **(N=49)** |
| --- | --- | --- | --- |
| **At Least One TEAE** | 15 (62.5) | 19 (76.0) | 34 (69.4) |
| **Blood and lymphatic system disorders** | 1 (4.2) | 1 (4.0) | 2 (4.1) |
| Leukocytosis | 1 (4.2) | 1 (4.0) | 2 (4.1) |
| **Cardiac disorders** | 1 (4.2) | 0 | 1 (2.0) |
| Atrial fibrillation | 1 (4.2) | 0 | 1 (2.0) |
| **Gastrointestinal disorders** | 4 (16.7) | 5 (20.0) | 9 (18.4) |
| Abdominal pain | 1 (4.2) | 0 | 1 (2.0) |
| Constipation | 0 | 2 (8.0) | 2 (4.1) |
| Diarrhea | 0 | 1 (4.0) | 1 (2.0) |
| Dyspepsia | 0 | 2 (8.0) | 2 (4.1) |
| Flatulence | 0 | 1 (4.0) | 1 (2.0) |
| Hematochezia | 1 1 (4.2) | 0 | 1 (2.0) |
| Nausea | 2 (8.3) | 2 (8.0) | 4 (8.2) |
| **General disorders and administration site conditions** | 1 (4.2) | 3 (12.0) | 4 (8.2) |
| Impaired healing | 0 | 1 (4.0) | 1 (2.0) |
| Necrosis | 0 | 1 (4.0) | 1 (2.0) |
| Pain | 0 | 1 (4.0) | 1 (2.0) |
| Pyrexia | 1 (4.2) | 1 (4.0) | 2 (4.1) |
| **Immune system disorders** | 0 | 1 (4.0) | 1 (2.0) |
| Drug hypersensitivity | 0 | 1 (4.0) | 1 (2.0) |
| **Infections and infestations** | 1 (4.2) | 2 (8.0) | 3 (6.1) |
| *Clostridioides difficile* infection | 0 | 1 (4.0) | 1 (2.0) |
| Osteomyelitis | 1 (4.2) | 0 | 1 (2.0) |
| Staphylococcal infection | 1 (4.2) | 0 | 1 (2.0) |
| Urinary tract infection | 0 | 2 (8.0) | 2 (4.1) |
| **Injury, poisoning and procedural complications** | 1 (4.2) | 0 | 1 (2.0) |
| Ligament sprain | 1 (4.2) | 0 | 1 (2.0) |
| **Investigations** | 8 (33.3) | 8 (32.0) | 16 (32.7) |
| Alanine aminotransferase increased | 1 (4.2) | 0 | 1 (2.0) |
| Aspartate aminotransferase increased | 1 (4.2) | 0 | 1 (2.0) |
| Blood alkaline phosphatase increased | 1 (4.2) | 0 | 1 (2.0) |
| Blood count abnormal | 0 | 1 (4.0) | 1 (2.0) |
| Blood creatinine increased | 1 (4.2) | 0 | 1 (2.0) |
| Blood fibrinogen increased | 0 | 1 (4.0) | 1 (2.0) |
| Blood lactate dehydrogenase increased | 2 (8.3) | 0 | 2 (4.1) |
| Blood lactic acid increased | 0 | 1 (4.0) | 1 (2.0) |
| Blood pressure orthostatic | 1 (4.2) | 0 | 1 (2.0) |
| C-reactive protein increased | 0 | 1 (4.0) | 1 (2.0) |
| Eosinophil count increased | 1 (4.2) | 0 | 1 (2.0) |
| Gamma-glutamyltransferase increased | 3 (12.5) | 0 | 3 (6.1) |
| Inflammatory marker increased | 1 (4.2) | 4 (16.0) | 5 (10.2) |
| Interleukin level increased | 1 (4.2) | 0 | 1 (2.0) |
| Liver function test increased | 1 (4.2) | 0 | 1 (2.0) |
| **Metabolism and nutrition disorders** | 4 (16.7) | 3 (12.0) | 7 (14.3) |
| Diabetes mellitus | 1 (4.2) | 0 | 1 (2.0) |
| Diabetic ketoacidosis | 0 | 1 (4.0) | 1 (2.0) |
| Failure to thrive | 0 | 1 (4.0) | 1 (2.0) |
| Fluid overload | 1 (4.2) | 0 | 1 (2.0) |
| Hyperglycemia | 1 (4.2) | 0 | 1 (2.0) |
| Hypernatremia | 1 (4.2) | 0 | 1 (2.0) |
| Hyperuricemia | 1 (4.2) | 0 | 1 (2.0) |
| Hypokalemia | 1 (4.2) | 0 | 1 (2.0) |
| Metabolic acidosis | 0 | 1 (4.0) | 1 (2.0) |
| **Musculoskeletal and connective tissue disorders** | 1 (4.2) | 0 | 1 (2.0) |
| Muscle spasms | 1 (4.2) | 0 | 1 (2.0) |
| **Nervous system disorders** | 4 (16.7) | 3 ( 12.0) | 7 (14.3) |
| Basal ganglia hemorrhage | 1 (4.2) | 0 | 1 (2.0) |
| Dizziness | 0 | 2 (8.0) | 2 (4.1) |
| Headache | 2 (8.3) | 1 (4.0) | 3 (6.1) |
| Metabolic encephalopathy | 0 | 1 (4.0) | 1 (2.0) |
| Tremor | 1 (4.2) | 0 | 1 (2.0) |
| **Psychiatric disorders** | 2 (8.3) | 1 (4.0) | 3 (6.1) |
| Delirium | 1 (4.2) | 0 | 1 (2.0) |
| Insomnia | 1 (4.2) | 1 (4.0) | 2 (4.1) |
| **Renal and urinary disorders** | 3 (12.5) | 0 | 3 (6.1) |
| Acute kidney injury | 3 (12.5) | 0 | 3 (6.1) |
| **Respiratory, thoracic and mediastinal disorders** | 2 (8.3) | 6 ( 24.0) | 8 (16.3) |
| Asthma | 0 | 1 (4.0) | 1 (2.0) |
| Cough | 0 | 1 (4.0) | 1 (2.0) |
| Dyspnea | 0 | 1 (4.0) | 1 (2.0) |
| Lung infiltration | 1 (4.2) | 0 | 1 (2.0) |
| Rales | 0 | 1 (4.0) | 1 (2.0) |
| Respiratory failure | 1 (4.2) | 4 (16.0) | 5 (10.2) |
| **Skin and subcutaneous tissue disorders** | 3 (12.5) | 1 (4.0) | 4 (8.2) |
| Acne | 1 (4.2) | 0 | 1 (2.0) |
| Decubitus ulcer | 0 | 1 (4.0) | 1 (2.0) |
| Pruritus | 1 (4.2) | 0 | 1 (2.0) |
| Rash | 1 (4.2) | 0 | 1 (2.0) |
| **Surgical and medical procedures** | 1 (4.2) | 0 | 1 (2.0) |
| Leg amputation | 1 (4.2) | 0 | 1 (2.0) |
| **Vascular disorders** | 1 (4.2) | 1 (4.0) | 2 (4.1) |
| Hypotension | 1 (4.2) | 0 | 1 (2.0) |
| Venous thrombosis limb | 0 | 1 (4.0) | 1 (2.0) |

**Supplemental Table 5**

| **System Organ Class Preferred Term, n (%)** | **Favipiravir+SOC**  **(N=24)** | **SOC**  **(N=25)** | **Overall**  **(N=49)** |
| --- | --- | --- | --- |
| **At Least One Serious TEAE** | 2 (8.3) | 3 ( 12.0) | 5 (10.2) |
| **General disorders and administration site conditions** | 0 | 1 (4.0) | 1 (2.0) |
| Necrosis | 0 | 1 (4.0) | 1 (2.0) |
| **Infections and infestations** | 1 (4.2) | 1 (4.0) | 2 (4.1) |
| *Clostridioides difficile* infection | 0 | 1 (4.0) | 1 (2.0) |
| Osteomyelitis | 1 (4.2) | 0 | 1 (2.0) |
| Staphylococcal infection | 1 (4.2) | 0 | 1 (2.0) |
| Urinary tract infection | 0 | 1 (4.0) | 1 (2.0) |
| **Metabolism and nutrition disorders** | 0 | 1 (4.0) | 1 (2.0) |
| Failure to thrive | 0 | 1 (4.0) | 1 (2.0) |
| **Nervous system disorders** | 1 (4.2) | 0 | 1 (2.0) |
| Basal ganglia hemorrhage | 1 (4.2) | 0 | 1 (2.0) |

**Supplemental Table 6. Favipiravir and T-705 M1 Plasma Concentration (µg/mL) over Time (Pharmacokinetic Population)**

| **Analyte** | **Sampling point** | **n** | **Mean (µg/mL)** | **SD (µg/mL)** | **SEM (µg/mL)** | **%CV** | **Geometric Mean (µg/mL)** | **Geometric %CV** | **Median (µg/mL)** | **Min (µg/mL)** | **Max (µg/mL)** |
| --- | --- | --- | --- | --- | --- | --- | --- | --- | --- | --- | --- |
| ***Favipiravir*** | Day1-Post | 21 | 34.0 | 16.5 | 3.60 | 48.6 | 29.6 | 63.7 | 32.70 | 6.31 | 69.6 |
|  | Day2-Pre | 22 | 14.3 | 16.3 | 3.48 | 114.1 | 6.9 | 276.6 | 11.00 | 0.149 | 67.8 |
|  | Day3-Post | 19 | 33.9 | 29.6 | 6.79 | 87.4 | 22.2 | 163.1 | 31.90 | 0.956 | 135 |
|  | Day8-Pre | 16 | 12.7 | 22.9 | 5.72 | 180.6 | 4.2 | 326.2 | 3.74 | 0.269 | 92.4 |
|  | Day11-Post | 19 | 42.4 | 27.2 | 6.24 | 64.1 | 35.2 | 75.5 | 35.20 | 5.42 | 130 |
|  | Day14-Pre | 13 | 21.3 | 25.4 | 7.05 | 119.3 | 11.8 | 205.1 | 15.30 | 0.855 | 100 |
| ***T-705 M1*** | Day1-Post | 21 | 15.1 | 7.2 | 1.57 | 47.5 | 13.4 | 59.7 | 15.40 | 2.76 | 36.6 |
|  | Day2-Pre | 22 | 4.4 | 3.3 | 0.69 | 73.8 | 3.2 | 123.8 | 3.54 | 0.261 | 14.9 |
|  | Day3-Post | 19 | 5.1 | 2.3 | 0.52 | 44.2 | 4.5 | 68.1 | 5.28 | 1.06 | 9.30 |
|  | Day8-Pre | 16 | 4.1 | 4.1 | 1.02 | 98.8 | 3.0 | 88.2 | 2.41 | 1.15 | 17.0 |
|  | Day11-Post | 19 | 6.5 | 4.0 | 0.92 | 62.1 | 5.7 | 49.9 | 5.61 | 2.27 | 20.8 |
|  | Day14-Pre | 13 | 5.8 | 5.2 | 1.45 | 90.1 | 4.5 | 77.7 | 3.79 | 1.91 | 19.4 |

Exclusion of missing data on sample collection Date/Time of the following.

ID 107-0002 on DAY3, ID 107-0004 on DAY1 POST, ID 107-0006 on DAY 8 and DAY 14, ID 107-0008 on DAY 14.

Subjects and sampling points for other analytical visits.

ID 103-0005 on DAY 8-POST, ID 103-0012 on DAY 3-PRE, ID 107-0004 on DAY 8-POST, ID 107-0009 on DAY 11-PRE.

**Supplemental Table 7. Plasma Pharmacokinetic Parameters of Favipiravir (Pharmacokinetic Population)**

| **Rel. Study Day [1]** | **Statistic** | **C_min_ (µg/mL)** | **C_max_ (µg/mL)** | **AUC_0-24h_ (µg*h/mL)** |
| --- | --- | --- | --- | --- |
| ***Favipiravir*** | | | | |
| 1 | N | 23 | 23 | 23 |
|  | Mean (SD, SEM) | 6.58 (8.56, 1.78) | 56.0 (16.9, 3.53) | 473 (252, 52.6) |
|  | %CV | 130.0 | 30.3 | 53.4 |
|  | Geometric Mean | 2.89 | 52.9 | 418 |
|  | Geometric %CV | 272.4 | 38.3 | 54.3 |
|  | Median (Min, Max) | 3.24 (0.120, 36.1) | 60.9 (22.0, 84.5) | 414 (161, 1240) |
|  |  |  |  |  |
| 2 | N | 23 | 23 | 23 |
|  | Mean (SD, SEM) | 13.3 (15.3, 3.19) | 46.8 (19.4, 4.04) | 619 (434, 90.5) |
|  | %CV | 115.1 | 41.4 | 70.1 |
|  | Geometric Mean | 7.03 | 43.1 | 509 |
|  | Geometric %CV | 223.1 | 44.4 | 70.9 |
|  | Median (Min, Max) | 8.19 (0.152, 70.2) | 47.1 (16.7, 109) | 488 (135, 2120) |
|  |  |  |  |  |
| 8 | N | 20 | 20 | 20 |
|  | Mean (SD, SEM) | 18.8 (18.6, 4.16) | 53.6 (24.4, 5.45) | 808 (519, 116) |
|  | %CV | 98.9 | 45.4 | 64.3 |
|  | Geometric Mean | 12.1 | 48.8 | 676 |
|  | Geometric %CV | 151.5 | 48.3 | 69.9 |
|  | Median (Min, Max) | 17.1 (1.04, 88.4) | 51.4 (17.1, 133) | 786 (189, 2580) |
|  |  |  |  |  |
| 14 | N | 17 | 17 | 17 |
|  | Mean (SD, SEM) | 18.0 (19.9, 4.83) | 52.4 (25.9, 6.29) | 800 (574, 139) |
|  | %CV | 110.4 | 49.5 | 71.7 |
|  | Geometric Mean | 10.5 | 46.6 | 641 |
|  | Geometric %CV | 187.1 | 56.9 | 82.7 |
|  | Median (Min, Max) | 15.5 (0.815, 87.4) | 50.8 (14.2, 134) | 772 (151, 2680) |
| ***T-705 M1*** | | | | |
| 1 | N | 23 | 23 | 23 |
|  | Mean (SD, SEM) | 3.78 (2.84, 0.593) | 18.7 (7.80, 1.63) | 245 (114, 23.7) |
|  | %CV | 75.1 | 41.8 | 46.3 |
|  | Geometric Mean | 2.87 | 17.2 | 225 |
|  | Geometric %CV | 97.6 | 45.0 | 42.5 |
|  | Median (Min, Max) | 3.44 (0.388, 11.7) | 16.4 (6.66, 37.3) | 209 (106, 559) |
|  |  |  |  |  |
| 2 | N | 23 | 23 | 23 |
|  | Mean (SD, SEM) | 3.84 (2.48, 0.517) | 8.82 (3.90, 0.812) | 155 (77.8, 16.2) |
|  | %CV | 64.6 | 44.2 | 50.2 |
|  | Geometric Mean | 3.11 | 8.17 | 140 |
|  | Geometric %CV | 81.9 | 40.1 | 49.3 |
|  | Median (Min, Max) | 3.26 (0.422, 10.9) | 7.54 (3.93, 19.0) | 127 (40.4, 374) |
|  |  |  |  |  |
| 8 | N | 20 | 20 | 20 |
|  | Mean (SD, SEM) | 4.83 (2.91, 0.650) | 8.63 (3.76, 0.841) | 163 (72.7, 16.3) |
|  | %CV | 60.2 | 43.6 | 44.5 |
|  | Geometric Mean | 4.18 | 8.02 | 152 |
|  | Geometric %CV | 58.9 | 38.6 | 39.3 |
|  | Median (Min, Max) | 4.36 (1.39, 13.5) | 7.40 (5.16, 18.9) | 145 (85.1, 390) |
|  |  |  |  |  |
| 14 | N | 17 | 17 | 17 |
|  | Mean (SD, SEM) | 4.77 (2.60, 0.631) | 9.01 (3.84, 0.930) | 172 (78.2, 19.0) |
|  | %CV | 54.5 | 42.6 | 45.6 |
|  | Geometric Mean | 4.20 | 8.36 | 159 |
|  | Geometric %CV | 56.1 | 39.8 | 40.5 |
|  | Median (Min, Max) | 4.35 (1.55, 12.1) | 7.80 (5.17, 17.9) | 153 (90.9, 391) |

C_min_ (average of across 2 dosing), C_max_ (average of across 2 dosing), AUC_0-24h_ (sum of across 2 dosing intervals)

Because the first administration time has two types of morning and night, each dosing date was defined as follows.

1st dose and 2nd dose as Day 1, 3rd dose and 4th dose as Day 2, 15th dose and 16th dose as Day 8, 27th dose and 28th dose as Day 15.

As an exception, ID 102-0004 used the 14th dose and 15th dose as Day 8, ID 107-0013 used the 13th dose and 14th dose as Day 8.

Replaced <0.02 µg/mL of C_min_ as 0.02 µg/mL.

*Programming Note: Report Mean (SD, SEM) with 3 significant digits, %CV with 1 decimal place, Geometric mean with 3 significant digits, Geometric %CV with 1 decimal place, and Median/Min/Max with 3 significant digits.*

Source Listing(s): 16.2.9.4.2; PROGRAM NAME: PRG140402
